# High-Performance Supercapacitors with Femtosecond-Laser-Nanostructured Current Collectors

**DOI:** 10.1021/acsaenm.5c00389

**Published:** 2025-11-03

**Authors:** Oleksandr Kuznetsov, Fedir Ivashchyshyn, Andriy Lotnyk, Nils Braun, Andrea Prager, Volodymyr Babizhetskyy, Anatoly V. Zayats, Iaroslav Gnilitskyi

**Affiliations:** † “NoviNano” Lab LLC, Pasternaka, 5, 79015 Lviv, Ukraine; ‡ Department of Applied Physics and Nanomaterials Science, 226328 Lviv Polytechnic National University, 12, Bandery Str., 79013 Lviv, Ukraine; § 28395 Leibniz Institute of Surface Engineering (IOM), Permoserstr. 15, D-04318 Leipzig, Germany; ∥ Department of Inorganic Chemistry, Ivan Franko National University of Lviv, 6 Kyryla i Mefodiya Str., 79005 Lviv, Ukraine; ⊥ Department of Physics and London Centre for Nanotechnology, 4616 King’s College London, Strand, WC2R 2LS London, U.K.

**Keywords:** supercapacitors, LIPSS, current collectors, femtosecond laser, electrodes

## Abstract

The interfacial resistance between a current collector and an active material of a supercapacitor leads to energy losses and a decrease in the specific power of the device. In addition, low adhesion of the active material to the collector can cause degradation of the supercapacitor during operation. In this study, we propose a method to reduce the interfacial resistance at the interface between a current collector and active material by forming laser-induced periodic surface structures (LIPSS). We show that laser structuring in inert gas (N_2_) environment results in improvement of electrochemical characteristics of supercapacitors. The charge-transfer resistance determined by the voltage drop during the cell discharge after femtosecond laser processing of the collectors decreases by 90% compared to the untreated collectors. The main effects of improving the electrochemical characteristics can be understood by an increase in the contact area between the electrode material and the collector due to the formation of LIPSS, which reduces the specific resistance. Laser structuring also causes certain chemical changes on the surface of the current collector, which can contribute to improving the electrical conductivity and the chemical stability of the contact. The LIPSS on the surface improves the adhesion of the active material to the current collector, which reduces the risk of mechanical delamination during the cyclic charge–discharge processes. A significant reduction in internal resistance with nanostructured electrodes opens promising avenues for increasing the specific power of supercapacitors of various types. The laser processing method does not require the use of additional reagents or multicomponent sublayers, which simplifies the approach and makes it attractive for application requiring scale up.

## Graphical abstract

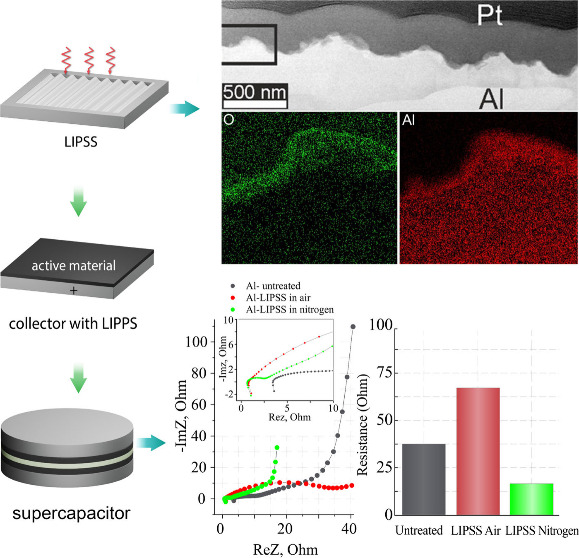

## 1 Introduction

The demand for high-efficiency energy storage systems has spurred the development of supercapacitors, which combine high energy and power densities. A critical component of these devices is the current collector, and various studies have shown that enhancing this element can significantly improve the overall performance of supercapacitors.^1,2^ Most research in this field focuses on advancing materials and processing methods to reduce electrical resistance, improve the adhesion of active materials, and ensure stability under harsh operating conditions. Metallic current collectors, such as aluminum, nickel, and copper, are widely used due to their high electrical conductivity and mechanical strength.^3,4^ However, these materials often face challenges, such as corrosion and high interfacial resistance. One solution involves chemical or laser etching, which creates micro- and nanostructures on the surface to increase the contact area and improve adhesion of active materials.^5–8^ Another method includes applying protective layers of graphite or other metals to reduce corrosion and enhance operational stability. The use of porous metals, such as metallic foams or three-dimensional structures, also lowers internal resistance and improves charge transport efficiency.^3,8^ For flexible electronic devices, metallic fabrics and films are being actively developed to combine high conductivity with flexibility.^9^ Carbon-based collectors, including graphene, carbon nanotubes, and carbon fibers, have also gained widespread use for their electrical conductivity, chemical stability, and lightweight.^10^ Surface modifications, such as the addition of functional groups, e.g., −COOH or −OH, enhance wettability and interaction with the electrolyte. Plasma treatment and laser irradiation help create rough and activated surfaces that increase the contact area.^7,11^ Effective approach involves coating carbon substrates with metallic layers to improve stability and conductivity. Carbon fabrics and three-dimensional structures allow the formation of porous surfaces that facilitate better ion transport.^7,12^ Polymer-based current collectors offer unique advantages such as flexibility, lightweight, and chemical stability, making them appealing for wearable devices.^13^ The use of composites enriched with carbon nanomaterials allows to achieve high conductivity.^14^ Such three-dimensional nanoporous structures fabricated from polymers significantly increase the contact area with the electrolyte.^15^ Integrating metal particles into polymer bases enhances adhesion and conductivity, while modifying paper-based current collectors with graphite or metals makes them cost-effective and lightweight solutions.^16^ Nevertheless, the intrinsic complexity of these methods brings about a barrier for large-scale applications in rechargeable commercial batteries.

Femtosecond laser structuring provides an efficient tool, offering a potentially maskless technology (single-step processing), allowing for a high-speed and low-cost patterning technique.^17^ Femtosecond pulses have also been exploited to induce self-organized periodic structures of submicron range in various materials.^18,19^ High-energy laser pulses can generate surface plasmon polaritons through plasma excitation even in dielectric materials, resulting in periodic laser-induced periodic surface structures (LIPSS) formed by the interference of surface polaritons.^20^ These self-organized periodic nanostructures have demonstrated potential in numerous fields, including sensors,^21^ photocatalysis,^22^ SERS^23^ and many others.^24–26^ The effect of LIPSS on the performance of supercapacitors and batteries remains largely unexplored.

In this paper, we study the effect of LIPSS created on the surface of aluminum current collectors using femtosecond laser structuring in different gaseous environments, including air and nitrogen. The effects of the environment on the structure formation, surface morphology, and chemical composition were investigated. The impact of LIPSS modifications of Al electrodes (Al-LIPSS) on the electrochemical performance, cyclic stability, and device efficiency was demonstrated. The developed approach offers new prospects for achieving efficient and resilient energy storage systems while ensuring ecological and economic viability.

## 2 Experimental Section

### 2.1 Femtosecond Laser Treatment

The aluminum foil samples were processed with a femtosecond Yb:KGW laser (Pharos, LightConversion) by raster scanning a laser beam with a wavelength of λ=1030 nm, power of 3.2 W, pulse energy of 16 μJ, repetition rate of 200 kHz. The step between the scan lines was 10 μm, and the scanning speed was 1 m/s. The diameter of the laser spot on the surface of the material in focus was 75 μm. Scanning mode with overlap 60% between pulses in one scanning direction (*c*) and with overlap between scanning lines (h) 50% was used for irradiation. Taking into account the overlap between pulses during scanning, approximately 112 pulses interacted with one point on the surface, and the fluence per pulse was 0.362 J/cm^2^. The foil was structured in either air or nitrogen gas environment using a gas cell (Figure S1 in the Supporting Information) with the same laser beam parameters (Table 1). These parameters provide the highest quality LIPSS in both air and nitrogen atmospheres. For treatment in nitrogen, gas was supplied at a pressure of 9 bar.

**Table 1:** Laser Parameters for Surface Treatment of Aluminum Foil for Current Collectors

λ, nm	Power, W	Energy, μJ	RR, kHz	Step, μm	Speed, m/s	Diameter, μm	Pulse per point	fluence, J/cm^2^
1030	3.2	16	200	10	1	75	112	0.362

### 2.2 LIPSS Characterization

The resulting LIPSS structure was investigated by scanning electron microscopy with an FEI Quanta 250 FEG ESEM. X-ray photoelectron spectroscopy (XPS) with a monochromatic X-ray source (Al–Kα; hυ = 1486.6 eV) was performed to determine the surface elemental composition. The optical properties, including absorbance and photoluminescence, were analyzed by using an Ocean Optics QE65Pro spectrophotometer. Diffuse reflectance measurements were conducted using an Ocean Optics QE PRO spectrometer coupled to an integrating sphere. A series of electrochemical tests was performed using a Gamry Reference 620 potentiostat.

Cross-sectional specimens for transmission electron microscopy (TEM) analysis were prepared by focused ion beam (FIB) (Auriga CrossBeam FIB-SEM, Carl Zeiss Microscopy GmbH). The TEM lamellae were cut with a Ga ion beam at an acceleration voltage of 30 kV. After the lift-out procedure, the lamellae were attached to TEM grids by local Pt deposition from a precursor and ion-milled to electron transparency with Ga ion beam. Pt protective layer was used for FIB lamellae preparations. TEM analysis was performed with a Titan3G2 instrument (FEI). The microscope was operated at a 300 kV acceleration voltages. TEM images were acquired in scanning TEM (STEM) mode using high-angle annular-dark-field (HAADF) and annular-bright-field (ABF) imaging conditions. EDX analysis was performed in STEM mode using a SuperX detector system.

The phase analysis of all specimens was performed by using powder X-ray diffraction data (PXRD). The PXRD intensity data were collected on an automatic diffractometer AXRD Proto (Cu Kα radiation, λ = 1.54185 Å, 2θ_max_ = 100°, step-scan mode with a step size of 0.015°(2θ) and a counting time of 25–30 s per data point, Si calibration external standard). In order to check the homogeneity of the samples and the textural effects Rietveld profile refinements of the X-ray powder pattern were performed using WinCSD software.^27^

X-ray photoelectron spectroscopy (XPS) analysis was carried out using an Axis Ultra instrument (Kratos Analytical, Ltd., Manchester, UK) with a monochromatic Al excitation source at 150 W (15 kV, 10 mA). The spectra were collected at a 160 eV pass energy with 1.0 eV nominal resolution. High resolution spectra were measured at a 40 eV pass energy with 0.1 eV nominal resolution.

### 2.3 Manufacturing of Electrode Components

Approximately 5% of the binder was added to the Norit Supra activated carbon to prevent the material from crumbling, and the mixture was then pressed into a thin film. The resulting carbon electrodes were bonded to the collector by using a conductive adhesive. As the binder, *N*,*N*-dimethylacetamide (DMAC) was applied. The adhesive was applied and spread onto the treated aluminum foil, which was subsequently placed in an oven to dry. To prepare the adhesive, the binder was dissolved in DMA, carbon black was added for conductivity, and the mixture was stirred until a thick, homogeneous paste was obtained.

Figure 1 shows the process of assembling the electrode pair. After nanostructuring of Al foil, a conductive adhesive was applied to the treated Al surface, and a carbon black film was pressed (a pressure of 5 tons at a temperature of 120°C for 1 min). It was cut to create an electrode. A 1 M aqueous solution of Na_2_SO_4_ electrolyte was used for electrical characterization. A total of 30 electrodes, each measuring 2 cm in diameter, were fabricated.

**Figure 1:**
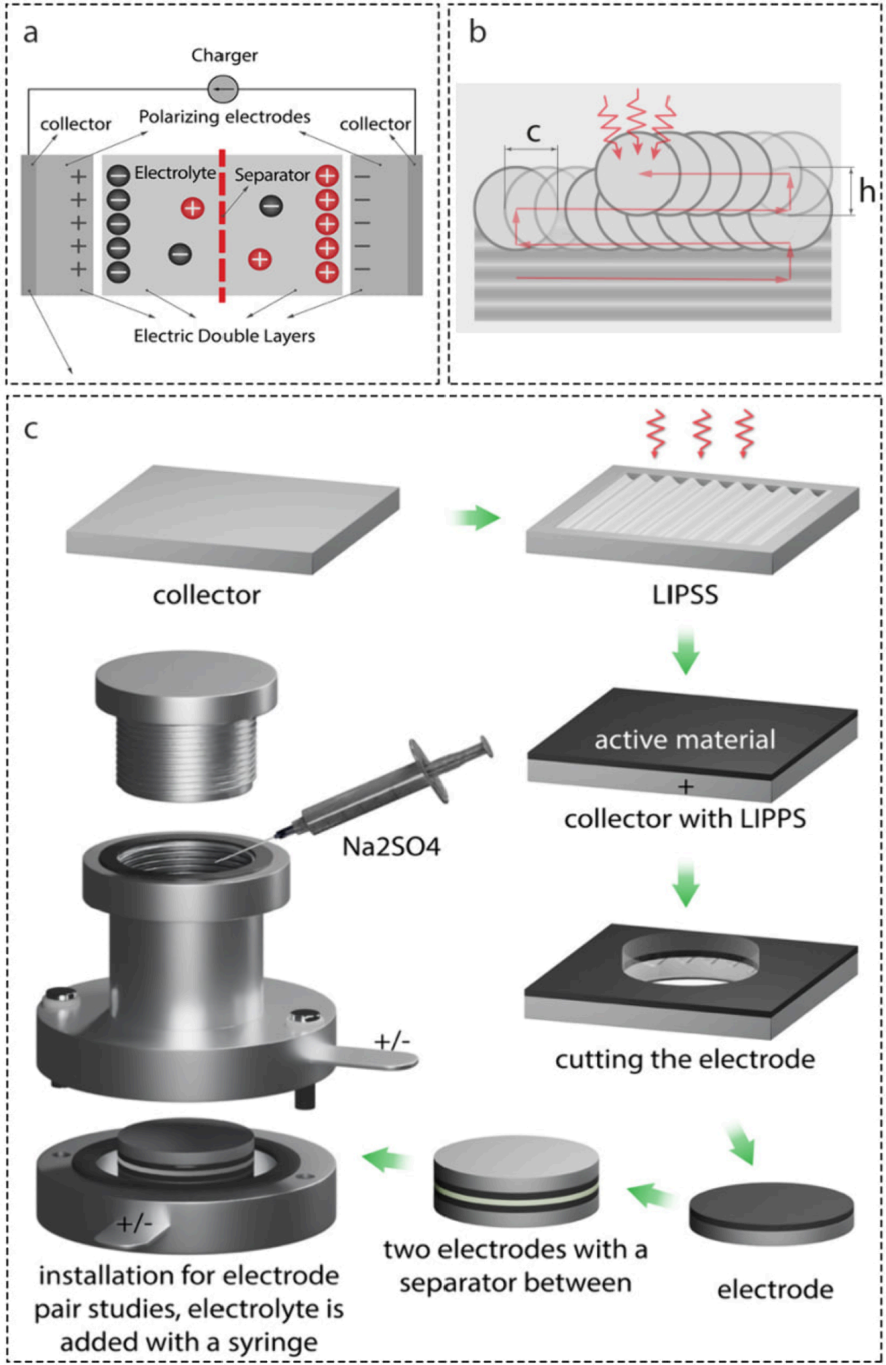
(a) Schematics of a symmetric supercapacitor. (b) Illustration of the scanning mode for laser treatment, showing a laser beam (circles) scanned in steps c and h in horizontal and vertical directions, respectively. (c) Workflow of fabrication of a supercapacitor cell.

The measurements were carried out under conditions as close as possible to those found in supercapacitors. For analysis, untreated aluminum foil and foil treated in air and nitrogen atmospheres were compared to assess the effect of the atmosphere on the formation of conductive or dielectric nanostructures on the surface. The experiments were conducted in a quick-assembly cell, and measurements were performed on a sufficiently large number of cells to ensure statistically reliable results, which were then summarized and presented in the manuscript. We believe that the cyclic stability demonstrated over 1000 cycles is reliable evidence of the stability of laser-treated collectors in this study. By continuing to cycle this type of electrochemical cell, we will observe more degradation processes of the electrolyte and active material, and therefore, it will be difficult to analyze the behavior of the aluminum collector itself.

## 3 Results and Discussion

### 3.1 Structural and Material Properties of Al-LIPSS

SEM images clearly show the formation of periodic nanopatterns on the surface of the aluminum foil, which is a characteristic feature of the formation of LIPSS (Figure 2). No significant melting or cracking of the Al surface was observed, which indicated optimal processing parameters. Wettability studies show that laser treatment resulting in nanostructure formation significantly affects the wettability of the electrodes with the electrolyte: the laser processing in air results in a wettability angle of 19.95° and in nitrogen 31.36°, compared to 114.79° for an untreated Al foil. The current results are not in line with the study,^28^ where hydrophobic surfaces showed better electrochemical results. However, in our case, the situation is substantially different. The electrodes in this research use activated carbon on laser-nanostructured aluminum current collectors. The key factor here is the contact resistance at the collector-electrode interface rather than ion adsorption alone. When the laser treatment makes the surface more hydrophilic, the electrolyte wets the surface more uniformly, which helps the carbon layer bond better and ensures more effective ion transport. This significantly reduces the interfacial resistance and improves the charge transfer. It is also important to note that many of the studies reporting advantages of hydrophobicity deal with carbon-based electrodes in organic electrolytes, where side reactions are a concern. In our aqueous electrolyte system with metallic collectors, good wettability actually helps performance by improving adhesion, reducing resistance, and stabilizing the electrode layer.

**Figure 2:**
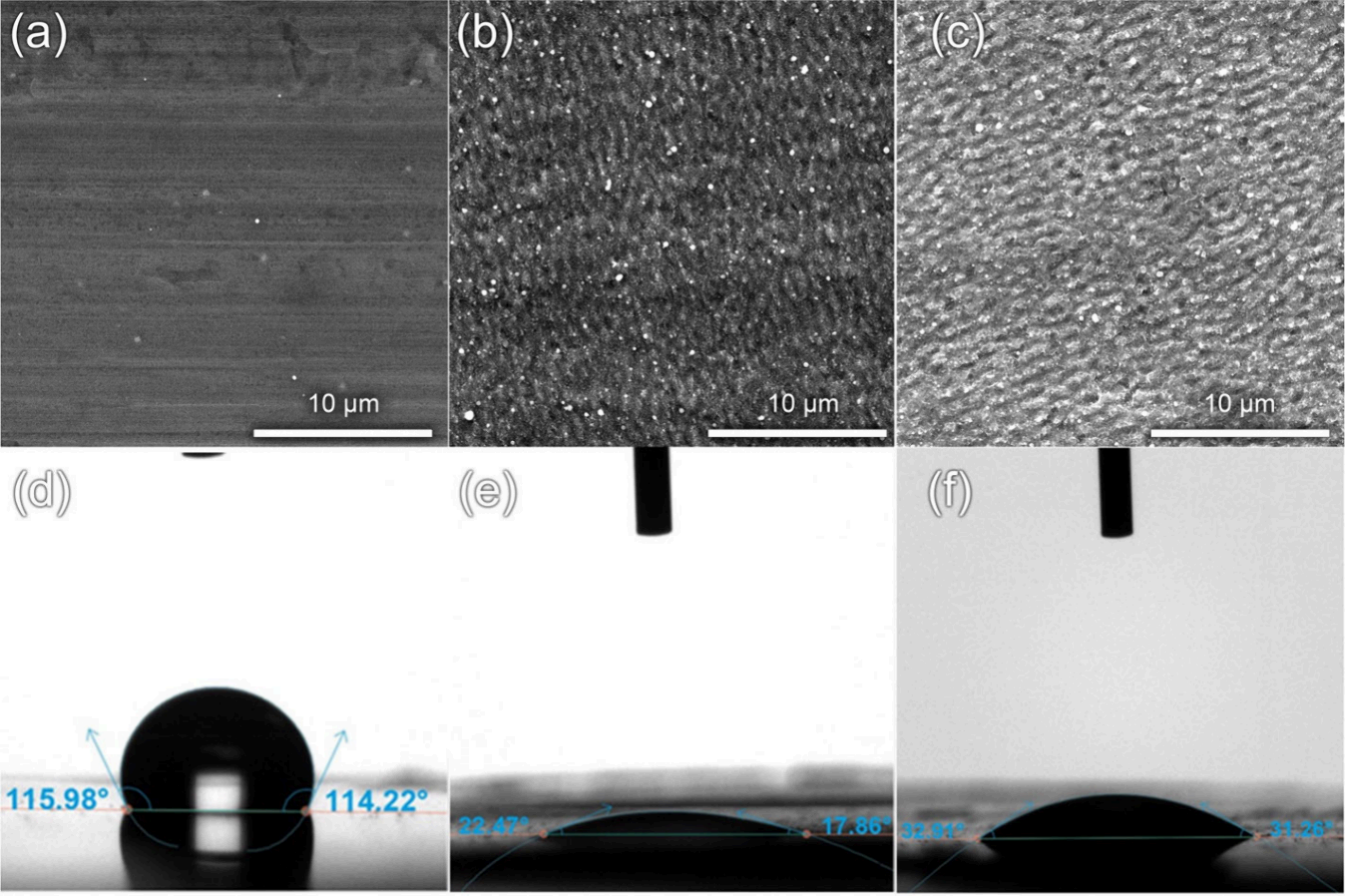
(a–c) SEM images and (d–f) electrolyte wettability angles of (a) untreated Al foil and after LIPSS formation in (b) air and (c) nitrogen.

The hydrophobic behavior is common for metals. The femtosecond laser treatment in air creates aluminum oxide (alumina, Al_2_O_3_) on the surface of Al, as was observed in TEM and XPS studies of Al-LIPSS discussed below. Alumina is a hydrophilic material. In nitrogen atmosphere, oxidation during the laser illumination is less pronounced, instead partial incorporation of nitrogen into aluminum subsurface takes place, which is seen in the increasing of the cell size of Al in the XRD results (Figure 2S in the Supporting Information). Therefore, the contact angle remains higher due to the lower surface polarity and reduced chemical affinity for water.

Figure 3 presents HAADF-STEM and ABF-STEM images of Al-LIPSS prepared in different environments. The images reveal the formation of a thick, nonuniform damaged layer in air-prepared LIPSS, with thicknesses ranging from 10 to 40 nm, along with cavities between the aluminum and the damaged layer. On top of this layer, dendrite-like structures up to 150 nm in length, as well as molten particles, are observed. EDX analysis confirms that both the dendrites and the damaged layer consist of aluminum oxide (Figure 4).

**Figure 3:**
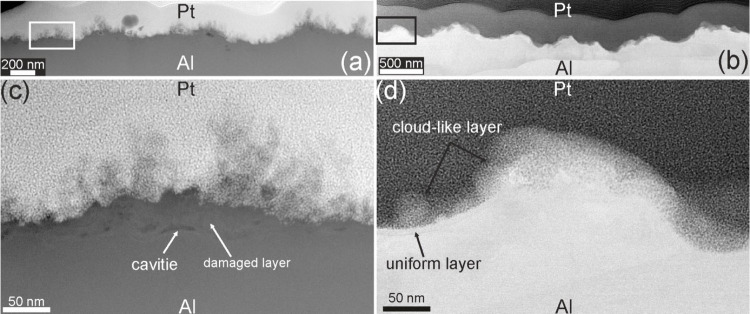
(a) Cross-sectional HAADF-STEM images of Al-LIPSS produced in air. (b) Cross-sectional ABF-STEM images of Al-LIPSS produced in nitrogen; (c,d) Magnified images of marked areas in (a) and (b) showing the details of surface structure.

**Figure 4:**
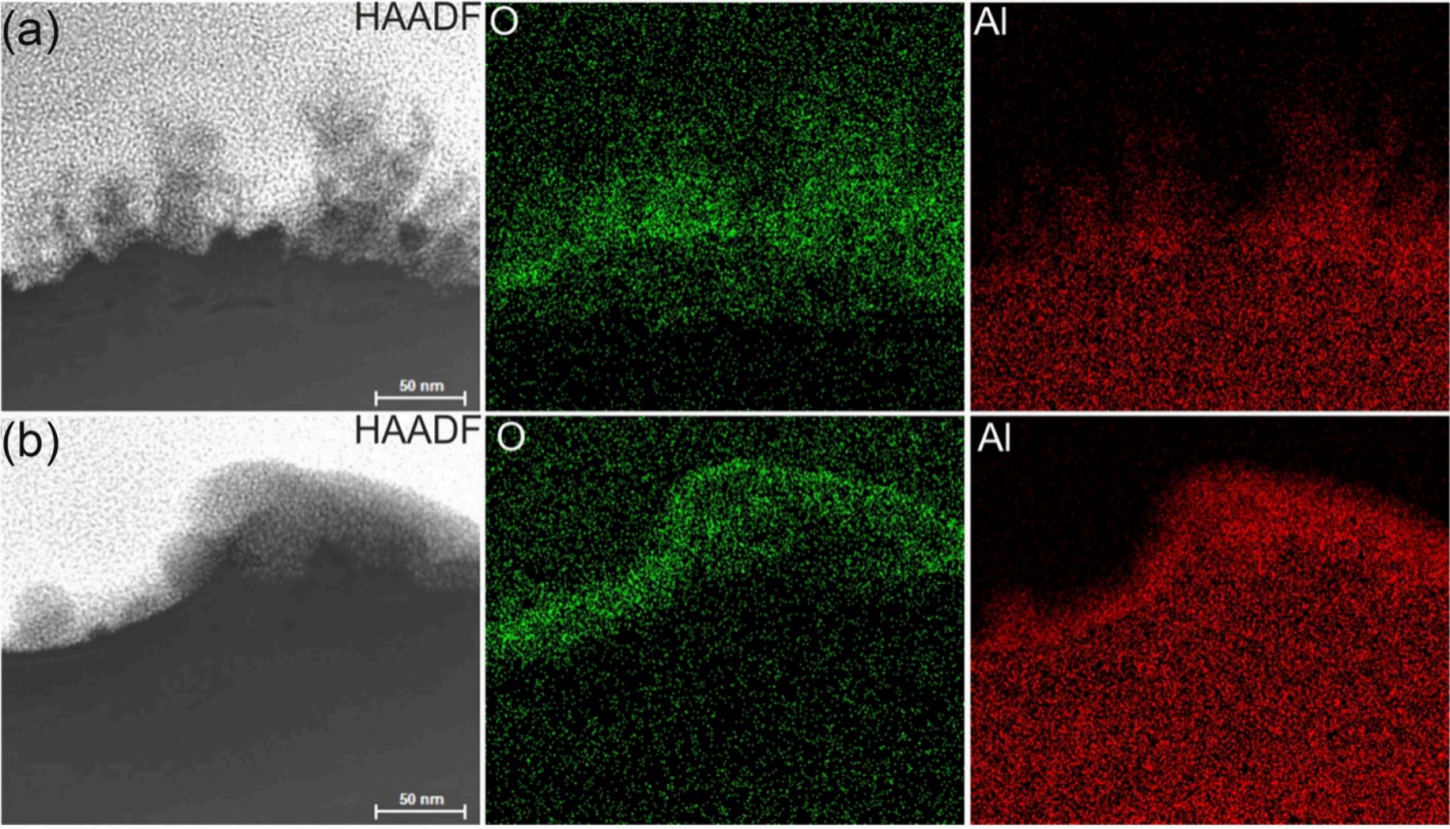
HAADF-STEM images with corresponding EDX-STEM elemental maps of Al-LIPSS prepared in (a) air and (b) nitrogen atmosphere.

In contrast, the surface of Al-LIPSS prepared in a nitrogen atmosphere exhibits a uniform amorphous layer of alumina with a thickness of approximately 6 nm, covering the entire surface. Additionally, a porous amorphous alumina layer with a nonuniform thickness ranging from 30 to 50 nm forms on top of the uniform layer, according to EDX mapping (Figure 4b).

To determine the lattice parameters and possible crystal structure transformations and structural effects on the surface, the whole pattern fitting of Rietveld refinement was applied to the acquired PXRD data for laser treated and untreated Al foils (Figure 2S in the Supporting Information). All PXRD patterns correspond to the cubic aluminum phase with some changes of lattice parameters from a = 4.0486 Å for untreated aluminum plate and laser-treated in air to a = 4.0521 Å for aluminum plate which was laser-treated in nitrogen. For the first two foils, the cell parameters correspond to pure aluminum.^29^ A small increase of the cell parameters for the laser-treated aluminum foil in nitrogen indicates some incorporation weak solubility of nitrogen in Al during laser treatment procedure.^30^ The XRD patterns from all aluminum foils show an intense [022] diffraction peak of aluminum due to the preferred crystallographic orientation of grains in a polycrystalline material. The refined coefficients of the preferred orientation using the March-Dollase function^31^ are 0.116 for untreated aluminum plate, 0.139 for laser-treated aluminum plate in air and 0.243 for laser-treated aluminum plate in nitrogen environment. The increase of the values indicates the decrease of the amount of the preferred plate orientation for the laser-treated aluminum plate surfaces in different gas environments.

The X-ray photoelectron spectroscopy (XPS) analysis further confirms the formation of aluminum oxide layers on Al-LIPSS surfaces (Figure 5). The Al oxide peak appears at 74.45 eV, while a second peak at 71.1 eV (Al-LIPSS in N) and 71.4 eV (for Al-reference) corresponds to metallic aluminum. Although the metallic peak is slightly shifted to lower energy, a similar peak at 71 eV has also been observed in the XPS spectra of aluminum foil surface, alongside an oxide peak at 74.1 eV.^34^ Moreover, the intensity of the metallic Al peak on Al-LIPSS prepared in air is very low, compared to the reference sample and Al-LIPSS in N, implying a much thicker Al oxide layer, in line with the TEM results. Furthermore, the XPS analysis reveals nitrogen incorporation into the surface of Al-LIPSS prepared in a nitrogen atmosphere, indicating to the formation of an Al–N complex (Figure 5b).

**Figure 5:**
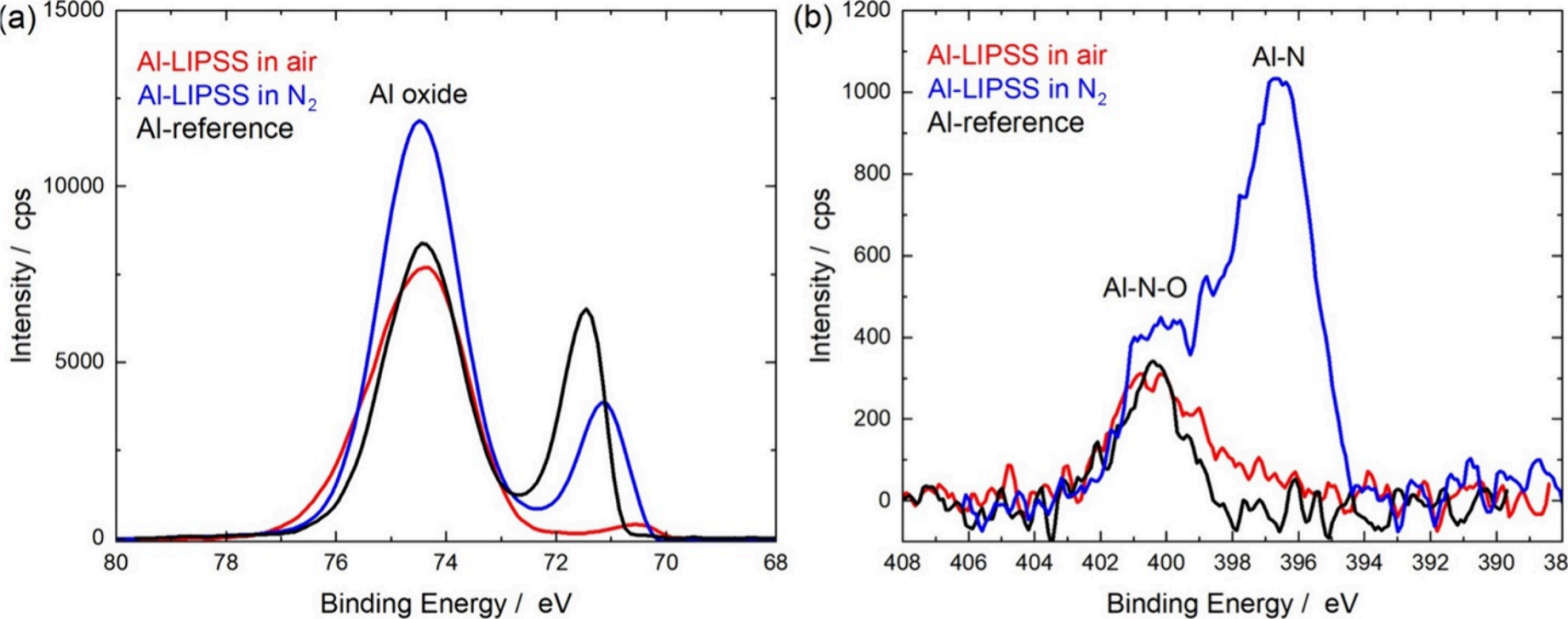
XPS spectra of laser treated and untreated aluminum foil surfaces: (a) core-level XPS spectra for Al-2p; (b) core-level XPS spectra for N-1s. The peak at a binding energy of 396.5 eV corresponds to Al–N bonds.^32^ Peaks at 400 eV are attributed to the binding energy of Al–N–O.^33^

The stronger Al oxide peak observed in the XPS spectra of the sample treated in a nitrogen atmosphere, compared to air, can be attributed to differences in oxidation dynamics and surface morphology induced by the laser-matter interaction under different ambient conditions. In air, the high oxygen concentration leads to rapid surface oxidation during laser treatment, resulting in the formation of dendritic Al structures, as confirmed by our TEM analysis. These features indicate nonuniform solidification and inhomogeneous oxide coverage, which reduces the consistency of the XPS signal from oxidized Al. In contrast, treatment in a nitrogen atmosphere suppresses immediate oxidation, allowing more uniform laser-induced melting and resolidification. Subsequent oxidation occurs more gradually and uniformly, leading to the formation of a continuous and homogeneous oxide layer, as also observed in the TEM images. This structural uniformity enhances the XPS signal, resulting in a stronger oxide peak. Thus, the higher Al oxide peak intensity in the nitrogen-treated sample reflects a more uniform surface oxide layer rather than a simple increase in oxide thickness.

### 3.3 Supercapacitor Performance

For performance testing, 20 electrodes with current collectors were fabricated, the surface of which was modified with a LIPSS in two different environments. The results of galvanostatic charge–discharge measurements for capacitors with differently modified electrodes are shown in Figure 6a. The charge/discharge cycles are characterized by flat lines, indicating the electrostatic accumulation of electric charge on the electrodes. From the obtained data, the main parameters of supercapacitor cells were determined as described in S1. Methods in the Supporting Information.

**Figure 6:**
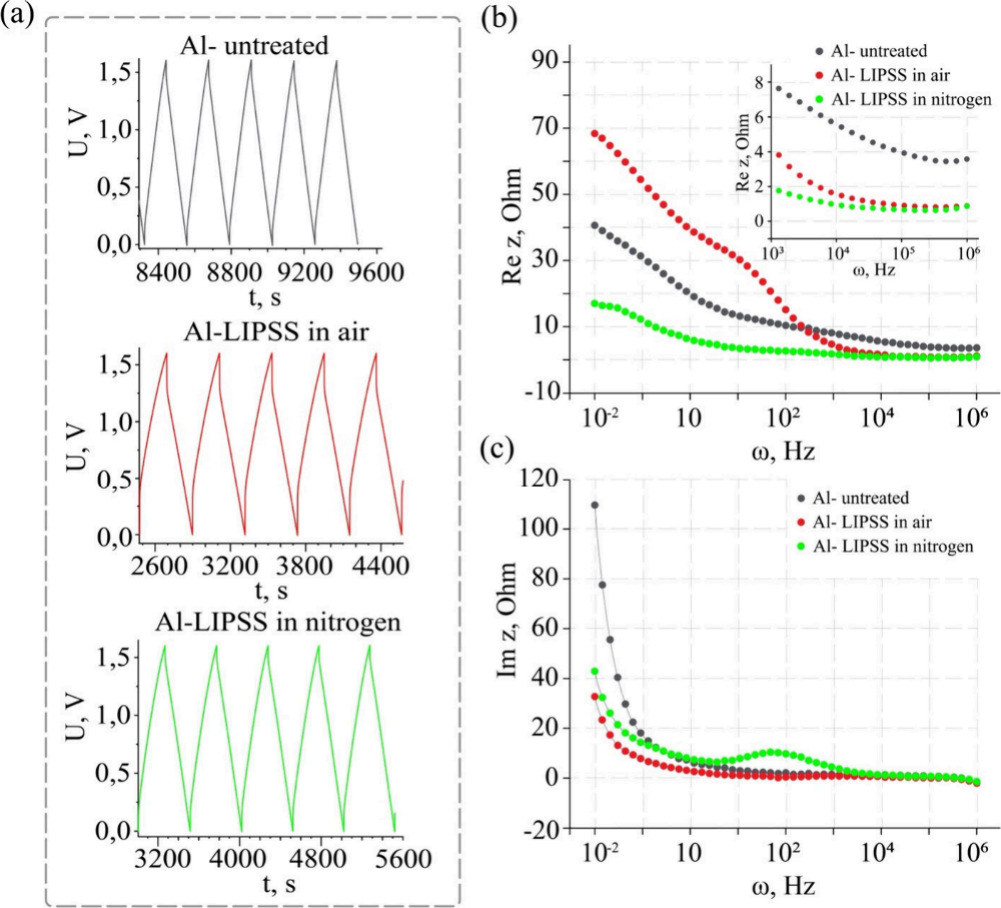
(a) Galvanostatic charge–discharge (GCD) profiles, (b) frequency dependences of the real component of complex impedance (Re­[Z]), and (c) frequency dependences of the imaginary component of complex impedance (Im­[Z]) for supercapacitor cells with untreated Al or Al-LIPSS current collectors.

The impedance dependences of the complex resistance on frequency measured for cells made of the same active carbon material but with different current collectors show frequency-dependent behavior observed for all types of electrodes. Re­[Z­(ω)] is monotonically decreasing, which reflects the main contribution to the charge transfer of the hopping conduction (Figure 6b). Given the amorphous and defective nature of the active carbon material, this is expected. The difference in Re­[Z] values depends on the current collector type used at low frequencies (>10^3^ Hz). The Al-LIPSS created in air has an additional barrier of a passivation layer of aluminum oxide for the transfer of charge carriers from the electrode to the collector. As frequency increases, the resistance decreases rapidly due to the activation of hopping conductivity because of the defective structure of the aluminum foil formed on the surface. At high frequencies, the resistance decreases by about 4 times compared to untreated foil, which can be caused by an increase in the surface area of the electrode and defects on its surface. In the case of treated foil in a protective nitrogen atmosphere, the opposite behavior is observed. In the low-frequency range, Re­[Z] has values lower than those of the untreated foil, which is due to the increase in the contact area of the current collector. No aluminum oxide dielectric film is formed, which would prevent the transfer of electric charge. At high frequencies, the resistance is more than 5 times lower than that of untreated foil.

The analysis of the behavior of the imaginary part of the complex resistance, Im­[Z­(ω)], shows that for both untreated electrodes and those treated in air, the same behavior is observed: a sharp decrease with increasing frequency (Figure 6c). Instead, for the Al-LIPSS electrodes obtained in a protective nitrogen atmosphere, the situation changes in the frequency range around ∼100 Hz, where a relaxation maximum is formed, indicating the formation of an additional structure with different properties compared to the amorphous carbon material. It can be assumed that a layer characterized by a semiconductor type of conductivity with an intrinsic relaxation time of charge carriers is formed on the aluminum surface, i.e., AlN.

The results of cyclic voltammetry show that the chemical activity of the Al electrode increases after the LIPSS formation as manifested in the appearance of maxima in the measured dependences (Figure 7a). These maxima indicate the occurrence of pseudocapacitive Faraday reactions of oxidation and reduction. This has also been confirmed from the Nyquist diagrams (Figure 7b). For all studied electrodes, inductive behavior at high frequencies is observed, which is expected given the amorphous and defective nature of the active carbon material. The supercapacitor made on the basis of a current collector made of untreated Al electrode shows the usual behavior of the complex impedance hodograph. The semicircle observed at high-frequencies indicates the processes of electric charge transfer through the carbon material particles. The semicircle at lower frequencies demonstrates the transfer of electric charge between the particles and the diffusion of electrolyte ions in the medium between them. When the Al electrode surface is treated with a femtosecond laser in air, the high-frequency resistance decreases, but both the high-frequency and low-frequency semicircles expand significantly. The treatment in nitrogen reduces both the high-frequency input impedance and the low-frequency impedance of the corresponding two semicircles.

**Figure 7:**
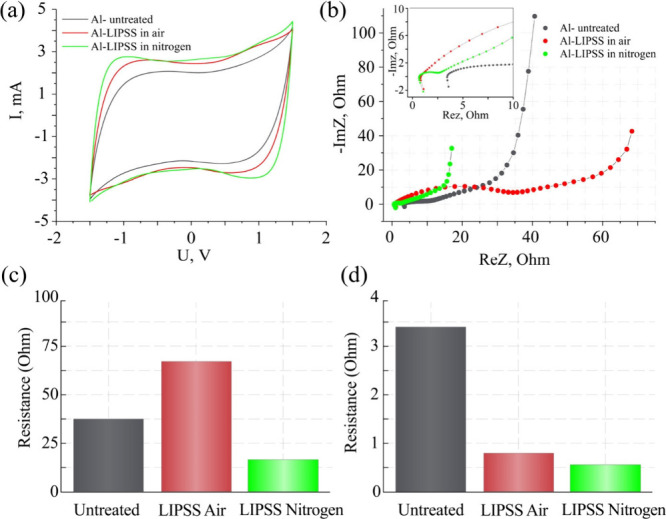
(a) Cyclic voltammograms measured for the supercapacitor cells with untreated and Al-LIPSS current collectors treated in different gas environments. (b) Nyquist diagram of the complex impedance for the supercapacitor cells with untreated Al and Al-LIPSS current collectors. (c) Resistance for the supercapacitor cell measured at constant current of the charge–discharge process. (d) Resistance for the supercapacitor cell measured at 300 kHz alternating current.

Based on the results, it can be concluded that treating the electrodes in air or nitrogen leads to a change in resistance during the charge–discharge process at a constant current (Figure 7c) and when measured at an alternating current of 300 kHz (Figure 7d), compared to the untreated surface. Based on the obtained data, averaged for each sample group, it is evident that the LIPSS samples treated upon nitrogen exhibits the lowest resistance at constant current of the charge–discharge process and resistance measured at 300 kHz alternating current (16.65 and 0.65 Ω), indicating improved conductivity. An energy efficiency in this case is 95.69%. LIPSS samples fabricated in an air environment exhibit much higher resistance at constant current of the charge–discharge process and exhibit relatively higher resistances measured at 300 kHz alternating current (69.15 and 0.83 Ω). However, they still provide a fairly high energy efficiency, LIPSS Air (95.28%). The untreated current collector exhibited an average resistance at constant current of the charge–discharge process and the highest resistance measured at 300 kHz alternating current (37.5 and 3.4 Ω). A energy efficiency in this case is 95.48%.

The change in conductivity of Al-LIPSS can be explained by the formation of periodic nanostructures that improve adhesion and change the transient resistance between the aluminum collector and the carbon active material layer. In the case of intense laser interaction with the surface, partial removal or restructuring of oxide layers may occur, which also affects the contact properties. Since the presence of oxides in many metals significantly increases interfacial resistance, minimizing these layers leads to improved current-carrying efficiency. By carefully selecting the parameters of laser treatment (wavelength, pulse repetition rate, pulse duration, and energy), it is possible to deliberately modify the surface structure and its chemical composition, as well as conductivity.

Testing of the supercapacitors demonstrates that employing LIPSS promotes a stronger adhesion of the active layer to the collector, which is important for the device long-term stability. In turn, improved contact between the active layer and the collector allows for more efficient energy transfer and reduced losses during the supercapacitor charge and discharge processes. Therefore, implementing LIPSS on metallic collectors is a promising approach to improving current collectors.

According to XPS and TEM, laser treatment in nitrogen atmosphere also results in the reduction of thickness of aluminum oxide layer, lowering electrical resistance and enhancing conductivity. Additionally, laser treatment significantly improved the wettability of the surface. The water contact angle decreased from 93.9° to 29.6°, promoting a uniform application of activated carbon and ensuring the stability of its layer. The electrochemical performance observed for supercapacitors with LIPSS-modified aluminum current collectors shows a notable improvement over untreated collectors and is comparable or superior to many results reported in the literature for metal-based EDLCs.^1,8,35–37^ After 1000 charge–discharge cycles, the modified supercapacitors retained initial parameters, demonstrating high stability. Furthermore, laser treatment improved the mechanical bonding between the current collector and the carbon layer, consistent with findings reported in previous study, where nanosecond laser structuring of metallic electrodes clearly improved adhesion.^38^ This work highlights that ultrafast laser pulse treatment is a promising, cost-effective, and environmentally friendly method for modifying current collectors for supercapacitors. Such modifications not only improve their electrochemical properties but also ensure the device stability and durability.

## 4 Conclusion

Femtosecond laser treatment of aluminum collectors was carried out in two environments (air and nitrogen), resulting in the formation of laser-induced periodic surface structures on the surface. Measurements of current–voltage characteristics and impedance revealed that the Al-LIPSS collectors exhibit significantly lower resistance compared to untreated aluminum. In particular, under constant-current charge–discharge conditions, the resistance was reduced by 85% with laser treatment in air and by 55% with treatment in nitrogen, relative to the untreated control sample. The charge transfer resistance also showed a substantial decrease of 76% with laser treatment in air and 80% with treatment in nitrogen. These results confirm the positive effect of laser modification on the conductivity of aluminum collectors, especially in a nitrogen environment, which demonstrated the best resistance reduction performance. The observed results can be explained by the fact that laser processing in an inert environment (nitrogen) occurs in a more controlled manner: unwanted oxidation processes are reduced, and the possible formation of nitride and oxynitride layers could improve the electrochemical properties of the collector. Improvements are also observed in air, although they are less pronounced.

Femtosecond LIPSS modification, especially under an inert nitrogen atmosphere, leads to lower interfacial resistance (due to oxide suppression and better contact, enhanced wettability promoting uniform electrode coating, improved mechanical bonding, and electrochemical stability). Laser treatment is not just a surface roughening but also tailors surface chemistry (e.g., oxide vs nitride formation) in a way that meaningfully impacts electrochemical behavior. LIPSS formation enhances surface area and adhesion, key factors in boosting both capacitance and durability. These modifications are scalable, cost-effective, and environmentally friendly, making them highly promising for next-generation energy storage device manufacturing.

The results of this study can be used to refine fabrication technologies for supercapacitor current collectors and other electrochemical energy storage devices, notably for increasing their longevity, specific capacitance, and energy efficiency. Elucidating the effects of different environments during laser processing makes it possible to better control the surface structure and chemical composition, paving the way for greater stability and standardization of the manufacturing process.

## Supplementary Material



## Notes

The definitions of the main parameters of the supercapacitors, the experimental setup for laser treatment of Al foils in a nitrogen gas environment, the X-ray powder diffraction patterns (XRD) and respective theoretical patterns for aluminum, and the XRD experimental patterns of aluminum foil surface (untreated, laser-treated in air and laser-treated in nitrogen) (PDF)
