# Supplementary material for: High-Performance Supercapacitors with Femtosecond-Laser-Nanostructured Current Collectors
Source: ACS Appl Eng Mater. 2025 Nov 3;3(11):3742–50. doi: 10.1021/acsaenm.5c00389 (PMC12670528; doi:10.1021/acsaenm.5c00389)
Supplement: Supplementary file 1 [file em5c00389_si_001.pdf]

Supporting information

High-performance supercapacitors with femtosecond-laser-nanostructured current collectors

Oleksandr Kuznetsov [†‡], *Fedir Ivashchyshyn* [‡], *Andriy Lotnyk* [§], *Nils Braun*[§], *Andrea Prager*[§], *Volodymyr Babizhetskyy* [¡], *Anatoly V. Zayats*[¿] *and Iaroslav Gnilitskyi*[*†‡¿]

[†]"NoviNano Lab" LLC, Pasternaka, 5, 79015 Lviv, Ukraine

[‡]Department of Applied Physics and Nanomaterials Science, Lviv Polytechnic National University, 12, Bandery Str., 79013 Lviv, Ukraine

[§] Leibniz Institute of Surface Engineering (IOM), Permoserstr. 15, D-04318 Leipzig, Germany

[¡] Department of Inorganic Chemistry, Ivan Franko National University of Lviv, 6 Kyryla i Mefodiya Str., 79005 Lviv, Ukraine

[¿] Department of Physics and London Centre for Nanotechnology, King's College London, Strand, WC2R 2LS London, UK.

**To whom correspondence should be addressed: iaroslav.gnilitskyi@kcl.ac.uk.*

S1. Methods

The main parameters of the fabricated supercapacitors, such as capacitance C, coulombic efficiency η, and internal resistance R, were determined from the obtained results of galvanostatic charging/discharging. These studies were carried out in the voltage range from 0 V to 1.6 V. This potential component was chosen from the point of view of electrolyte stability.

The capacity C was calculated using

$$C = \frac{2\, I\, t_d}{m_{ac}\, U_{max}} \tag{S1}$$

where I is the current strength at which the discharge occurred, t_d is the discharge time, m_{ac} is the weight of the active substance $m_{ac} = m_{el} - m_{bn}$, m_{el} is the weight of the electrode, m_{bn} is the weight of the binder (5% of the mass of the electrode), U_{max} is the voltage to which the two-electrode cell was charged.

The Coulomb efficiency η was calculated using

$$\eta = \frac{Q_{dis}}{Q_{chr}} \tag{S2}$$

where $Q_{dis} = I_{dis}\, t_{dis}$ is the total amount of charge extracted during discharge, $Q\_{chr} = I_{chr}\, t_{chr}$ is the total amount of charge supplied during charging, I_{dis}/I_{chr} represents the discharge/charge current, t_{dis}/t_{chr} is the discharge/charge duration.

The resistance R was determined from

$$R = \frac{\Delta U}{2I} \tag{S3}$$

where $\Delta U = U_{max} - U_{min}$ is the voltage drop during cell discharge, determined by the difference between U_{max} is the voltage to which the cell was charged, and U_{min} can be determined by the intersection of two lines, one drawn through the discharge points, and the other vertically through the maximum voltage.

The stability of this supercapacitor cell was tested using cyclic voltammetry. The stability of this supercapacitor cell was tested by cyclic voltammetry in the range from -1.5 V to +1.5 V and a potential sweep rate of 50 mV/s.

The impedance spectroscopy method was used to study the conductivity of this system in more detail. The impedance measurements of the experimental electrochemical cells of supercapacitors were carried out in the frequency range of $10^{-2} \div 10^{6}$ Hz using AUTOLAB PGSTAT-100 (Metrohm AG, Herisau, Switzerland), equipped with computer programs FRA-2 and GPES. The amplitude of the measuring signal was $5 \cdot 10^{-3}$ V.

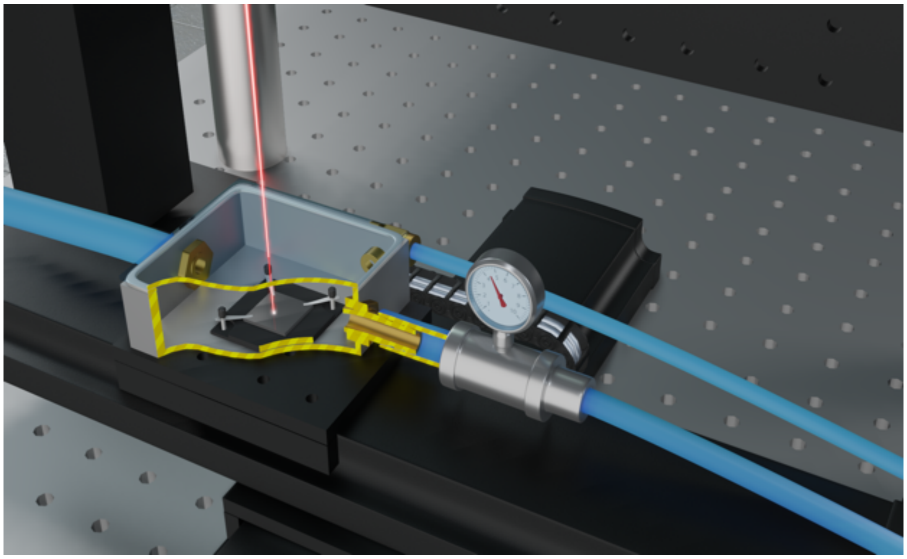

Figure S1. Experimental setup to laser treatment of Al foil in nitrogen gas environment.

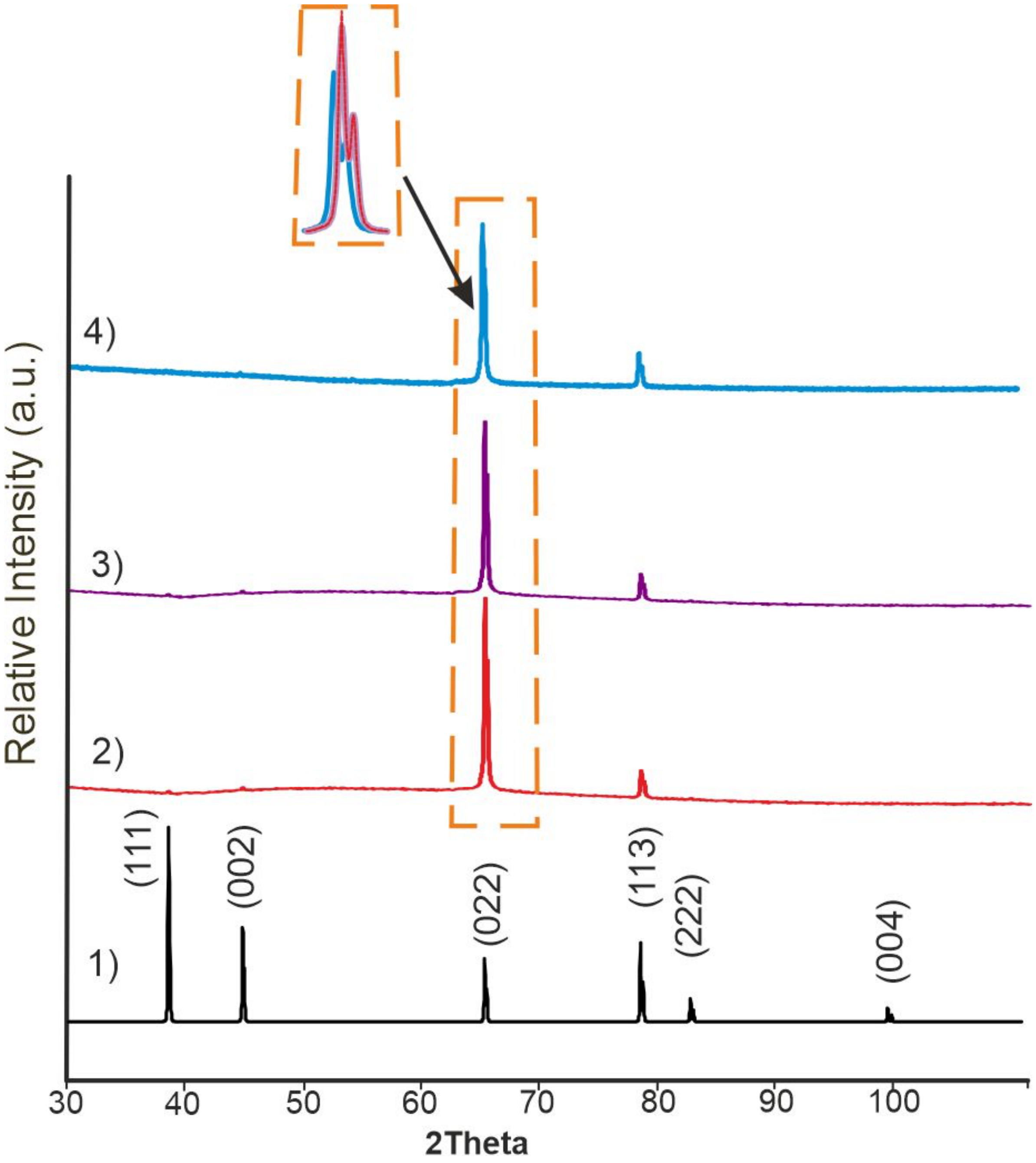

Figure S2. XRD theoretical pattern of aluminium (1) and XRD experimental patterns of aluminium foil surface: (2) untreated, (3) laser-treated in air; (4) laser-treated in nitrogen. Diffraction peaks of aluminium are marked. Inset shows the superposed different intensities and a 2Θ shift of textured [022] peaks for all Al foils.
